# Elevated Transaminases as Predictors of COVID-19 Pneumonia Severity

**DOI:** 10.3390/medicina58070842

**Published:** 2022-06-23

**Authors:** Tijana Radonjić, Ognjen Milićević, Igor Jovanović, Marija Zdravković, Marija Dukić, Olga Milorad Mandić, Jelica Bjekić-Macut, Olivera Borko Marković, Zoran Todorović, Milica Brajković, Novica Nikolić, Slobodan Klašnja, Višeslav Popadić, Anica Divac, Milica Marinković, Nabil Alhayek, Marija Svetislav Branković

**Affiliations:** 1 University Hospital Medical Center Bežanijska Kosa, 11000 Belgrade, Serbia; igordusanov@yahoo.com (I.J.); sekcija.kardioloska@gmail.com (M.Z.); mdukic107@gmail.com (M.D.); olga.rnjakovic@gmail.com (O.M.M.); jbjekic@yahoo.com (J.B.-M.); markovic.olivera@bkosa.edu.rs (O.B.M.); zoran.tdrvc@gmail.com (Z.T.); brajkovic.milica@yahoo.com (M.B.); novica.nikolic87@yahoo.com (N.N.); slobodan.klasnja@gmail.com (S.K.); viseslavpopadic@gmail.com (V.P.); anicadivac92@gmail.com (A.D.); marijasbrankovic@gmail.com (M.S.B.); 2 Faculty of Medicine, Institute for Medical Statistics and Informatics, University of Belgrade, 11000 Belgrade, Serbia; ognjen011@gmail.com; 3 Faculty of Medicine, University of Belgrade, 11000 Belgrade, Serbia; milicamarinkovic997@gmail.com (M.M.); nabilhayekk@gmail.com (N.A.)

**Keywords:** liver, transaminases, COVID-19, SARS-CoV-2

## Abstract

Background: This study aimed to calculate the frequency of elevated liver enzymes in hospitalized patients with coronavirus disease 2019 (COVID-19) infection and to test if liver enzyme biochemistry levels on admission could predict the computed tomography (CT) scan severity score of bilateral interstitial pneumonia. Methods: This single-center study comprised of 323 patients including their demographic data, laboratory analyses, and radiological findings. All the information was taken from electronic health records, followed by statistical analysis. Results: Out of 323 patients, 115 of them (35.60%) had aspartate aminotransferase (AST) and/or alanine aminotransferase (ALT) over 40 U/L on admission. AST was the best predictor of CT scan severity score of bilateral interstitial pneumonia (R^2^ = 0.313, Adjusted R^2^ = 0.299). CT scan severity score in the peak of the infection could be predicted with the value of AST, neutrophils, platelets, and monocytes count (R^2^ = 0.535, Adjusted R^2^ = 0.495). Conclusion: AST, neutrophils, platelets, and monocytes count on admission can account for almost half (49.5%) of the variability in CT scan severity score at peak of the disease, predicting the extensiveness of interstitial pneumonia related to COVID-19 infection. Liver enzymes should be closely monitored in order to stratify COVID-19 patients with a higher risk of developing severe forms of the disease and to plan the beforehand step-up treatment.

## 1. Introduction

According to World Health Organization statistics, by June 2022 there were over 532 million confirmed cases of coronavirus disease 2019 (COVID-19) and more than 6 million confirmed deaths [1]. The health, social, and economic consequences of this pandemic on the population are enormous and have created a worldwide tragedy [2]. Even after two years, the global severe acute respiratory syndrome coronavirus 2 (SARS-CoV-2) pandemic does not wane and remains a huge medical challenge. Although we know that most patients do not require hospital treatment, the rest could, at some point, require hospital, or even intensive care [3]. Most often, the patient presents with respiratory tract symptoms, and further diagnostic procedures frequently reveal viral pneumonia. This condition can lead to respiratory failure, the most severe manifestation of COVID-19, which requires mechanical ventilation. Unfortunately, the progression from noninvasive mechanical ventilation (NIV) to invasive mechanical ventilation (IMV) is associated with significantly higher morbidity and mortality [4]. 

On the other hand, COVID-19 is a systemic disease with multiple organ damage, liver injury being the most common after lungs [5,6]. This infection can increase liver enzymes in 15% to 53% of patients [5]. It is still unclear what is the underlying pathophysiology of liver damage, but it clearly has a multifactorial mechanism.

As the incidence of non-alcoholic fatty liver disease (NAFLD) is galloping, it is crucial to focus on it as an emerging issue, since it could progress to non-alcoholic steato-hepatitis (NASH) which can be a further predisposing factor for chronic liver complications [7]. NAFLD frequently overlaps with one or more elements of metabolic syndrome including obesity, insulin resistance, dyslipidemia, and arterial hypertension [8], which all are known to have a significant impact on the severity of COVID-19 infection.

We conducted a retrospective cohort analysis of liver injury seen in COVID-19 infection and investigated if liver damage interferes with the severity of the disease. The aim of this study was to calculate the frequency of elevated liver enzymes in hospitalized COVID-19 patients, to test if there is a correlation between liver injury and age, gender, hospital stay or computed tomography (CT) scan severity score, and to test if liver enzyme biochemistry levels on admission can predict CT scan severity score of bilateral interstitial pneumonia.

## 2. Materials and Methods

This single-center study included 323 COVID-19 patients hospitalized at University Hospital Centre Bežanijska kosa, Belgrade, Serbia, between June 2020 and August 2020. Our hospital was a reference COVID-19 center with more than 1000 COVID-19 patients per month and more than 100 patients treated in intensive care unit (ICU) by October 2020 [9]. Inclusion criteria were confirmed COVID-19 infection by antigen or polymerase chain reaction (PCR) test for SARS-CoV-2 or COVID-19 pneumonia seen on X-ray or CT scan of the thorax. We excluded all patients who were treated for COVID-19 before hospital admission, and all patients who had chronic liver diseases (including confirmed viral hepatitis infections), all hepatic autoimmune diseases, or liver insufficiencies. Additionally, we excluded patients who had elevated liver enzymes before admission and patients with diagnosis of non-alcoholic fatty liver disease (NAFLD) in their health records. All the data were collected retrospectively from electronic health records, including the demographic data, clinical data regarding oxygen support and use of mechanical ventilation, and laboratory results, more precisely complete blood count, C reactive protein (CRP), aspartate aminotransferase (AST), alanine aminotransferase (ALT) and D-dimer. We also included data regarding the diagnostic procedures such as lung X-ray and thorax CT scan.

### 2.1. Radiological Criteria

On admission, every patient was examined with chest X-ray, and additional ones were carried out for control purposes. Furthermore, CT scan of thorax was mandatory on admission to gain additional information regarding pneumonia severity. Characteristic findings on CT scan of thorax included ground-glass opacities, crazy paving patterns and thickening of the interlobular septum. CT severity score was calculated for every patient. Each lung lobe could score 0–5 points in accordance with the degree of extension of interstitial pneumonia in each. The minimum score for each lobe was 0 and the maximum was 5, which implies that the maximum CT severity score for both lungs is 25 (5 lung lobes with maximum involvement) [10]. These scores could be divided into mild pneumonia (scores 1–8), moderately severe (scores 9–16) and severe (scores 17–25). Moreover, interstitial COVID-19 pneumonia seen on a CT scan of the thorax was divided into 4 stages: early, progressive, peak, and resolution [10]. Additional CT scans were not performed routinely and only if necessary (in cases when serious pneumonia progression was suspected).

### 2.2. Laboratory Analyses

On admission, the complete blood count, C reactive protein, liver transaminases, and D-dimer were obligatorily determined, with our study emphasizing the parameters of liver function. The results were interpreted according to the reference values; for transaminases, cut-off value was 40 U/L, as per European Association for the Study of the Liver guidelines. Serology for hepatotropic viruses (hepatitis C virus, hepatitis B virus) was not performed, but patients who previously had any of these infections were excluded from the study.

### 2.3. Therapy

Patients were treated per recommendations of the National Guide for the Treatment of COVID-19 infection, which were based on the guidelines of the World Health Organization, and in accordance with the availability of drugs in Serbia. Antiviral therapy (Favipiravir) was administered in the first 5 to 7 days of the disease if liver transaminases were below 300 U/L as Favipiravir had shown hepatotoxicity. On the first day Favipiravir was given in a dose of 1600 mg two times a day, and afterwards for four more days in a dose of 600 mg two times a day. On admission, antibiotics were administered for prophylaxis, but subsequently changed based on antibiogram or empirically. Biological therapy was also available for administration and included mainly Tocilizumab (8 mg/kg, with a maximum dose of 2 × 800 mg). All patients received a prophylactic dose of low-molecular-weight heparin, while those with an increased risk of thrombosis and thromboembolic events received a therapeutic dose. Depending on the need, patients received oxygen support of various modalities (nasal cannula, standard mask, non-re-breathing mask, Hi-flow ventilation, non-invasive ventilation or mechanical ventilation).

### 2.4. Statistics

Categorical variables were described as frequency and percentages, and continuous variables as mean and SD, or median and IQR. Means for continuous variables were compared using independent group t-tests when the data were normally distributed; otherwise, median test was used. Comparison of categorical variables was carried out using the χ^2^ test or the Fisher exact test, if the cell counts were small. Multiple ordinary least square regression was used to explore the association between liver test abnormalities and the severity of the disease. All statistical analyses were performed using SPSS version 21.0 software (IBM Corp. Released 2012. IBM SPSS Statistics for Windows, Version 21.0. IBM Corp., Armonk, NY, USA). A 2-sided α of less than 0.05 was considered statistically significant.

## 3. Results

This study included 323 patients, out of which 185 were male (57.3%) and 138 were female (42.7%). The youngest patient was 21 years old while the oldest was 93 years old, with an average age of 57.32 ± 13.77 years. The average hospital length of stay was 12.85 ± 7.40 days, with the minimum hospital length of stay two days and maximum 56 days.

The average value of AST on admission was 37.57 ± 25.44 U/L, with the minimum value of 12 U/L and the maximum value of 201 U/L. Furthermore, the average value of ALT was 36.95 ± 28.18 U/L, with the minimum value of 7 U/L and the maximum value of 218 U/L. Moreover, out of 323 patients, 115 (35.60%) had AST and/or ALT over 40 U/L on admission. Out of 115 patients, 25 had only AST elevated (21.74%), while 26 had only ALT elevated (22.61%), with 64 patients who had both enzymes elevated (55.65%). It is important to emphasize that there was no fatal outcome in both groups (with and without elevated liver enzymes). Additionally, elevated liver enzymes were more frequently seen in male patients (76 out of 185 or 41.1%) compared to 28.3% of female patients (39 out of 138), and there was statistically significant difference between elevated liver enzymes and gender (*p* = 0.017). Moreover, the average age of patients with elevated liver enzymes was 55.97 ± 13.12 years. We investigated if there was a correlation between the age of patients with and without elevated transaminases, but there was no correlation found (*p* = 0.174). Additionally, the average hospital length of stay of patients with elevated liver enzymes was 13.53 ± 8.87 days, while it was 12.48 ± 6.57 days in those without. We also compared to see if there was a correlation between those two, but there was no statistical significance (*p* = 0.648).

**Figure 1:**
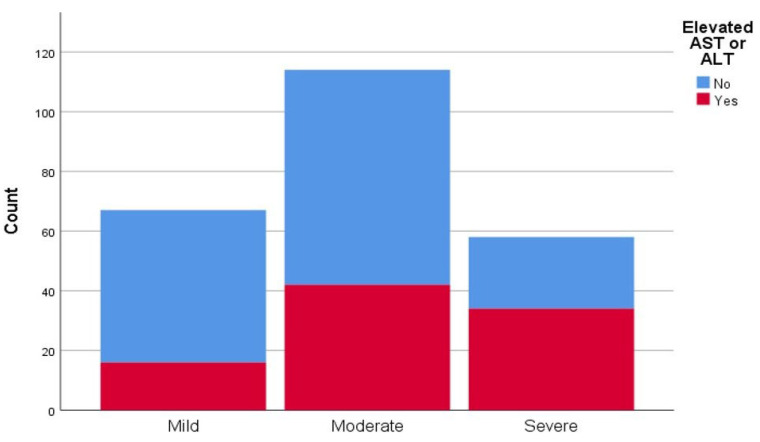
Representation of computed tomography (CT) scan severity score of interstitial pneumonia in patients with and without elevated liver transaminases (aspartate aminotransferase (AST), alanine aminotransferase (ALT).

The average value of CT scan severity score was 11.83 ± 6.20, with a median value of 11. There were 67 patients (28.03%) with mild pneumonia (CT scan severity scores 1–8), 114 (47.7%) with moderately severe pneumonia (CT scan severity scores 9–16), and 58 (24.27%) with severe pneumonia (CT scan severity scores 17–25). There were 16 patients (23.9%) with elevated liver enzymes who had mild pneumonia, 42 (36.8%) with moderately severe pneumonia and 34 (58.6%) with severe pneumonia (Figure 1). Furthermore, there was a correlation between patients who had elevated transaminases and their respective CT scan severity score, in contrast to patients without elevated transaminases (*p* < 0.005).

On the other hand, CT scan severity score was in correlation with the values of AST (ρ = 0.317, *p* < 0.01), ALT (ρ = 0.157, *p* = 0.015), gamma-glutamyl transferase (GGT) (ρ = 0.209, *p*-0.01) and CRP (ρ = 0.393, *p* < 0.01), but it was not in correlation with D-dimer (ρ = 0.94, *p* = 0.147). More patients on oxygen supply also had elevated liver enzymes, over 40 U/L (39.8%), compared to those without oxygen supply (23.2%). Further, patients who were treated with tocilizumab more frequently had AST and/or ALT over 40 U/L (52.8%), compared to patients that were not treated (32.2%).

In this study, we also tested regression of CT scan severity score of bilateral interstitial pneumonia, and the result indicated that the value of AST was the best single predictor of this (R^2^ = 0.313, Adjusted R^2^ = 0.299). We could predict CT scan severity score in patients with bilateral interstitial pneumonia with the value of AST and leukocytes and neutrophils count (R^2^ = 0.266, Adjusted R^2^ = 0.252) (Table 1). Additionally, CT scan severity score at peak of infection could be predicted by the value of AST and platelets and leukocytes count (R^2^ = 0.475, Adjusted R^2^ = 0.442). More significantly, we could predict CT scan severity score at peak of infection with the value of AST, neutrophils, platelets and monocytes count (R^2^ = 0.535, Adjusted R^2^ = 0.495) (Table 2). Moreover, patients with CT scan severity score at peak of infection more frequently had elevated liver enzymes, more precisely 40.8%, while on the other hand 36.9% of patients with CT scan severity score in non-peak state had elevated AST and/or ALT over 40 U/L.

## 4. Discussion

In all the reviewed literature, there are data that show that SARS-CoV-2 directly or indirectly causes liver damage, i.e., causes elevated liver transaminases [5,6]. There are some studies that investigated if liver enzymes could be a prognostic factor in this infection [11]. In the present study, we tested if liver laboratory parameters could predict CT scan severity score, which is the equivalent of the extensiveness of interstitial pneumonia. We observed a significant correlation between patients who had elevated transaminases and their respective CT scan severity score, in contrast to patients without elevated transaminases (*p* < 0.005). The most significant predictors in the study were the levels of AST (which is the best single predictor), neutrophils, platelets, and monocytes on admission and these can account for almost half (49.5%) of the variability in CT scan severity score at peak of the disease, which means we can in advance predict the extensiveness of interstitial pneumonia in the severe forms of COVID-19 related pneumonia.

In the study by Medetalibeyoglu et al. on 554 patients, 153 patients had elevated liver enzymes (27.62%) [11] in comparison to our study which had more patients with elevated liver transaminases (35.29%). In the aforementioned study, there was no statistically significant difference between age distribution among patients with and without elevated liver enzymes (AST and/or ALT > 40 U/L) [11]. Similar results were observed in our study (*p* = 0.174). Moreover, in this study, hospital length of stay was not statistically significantly longer in those with liver damage compared to those without it (*p* = 0.648). The median hospital length of stay in both groups was 11 days, with a minimum of 2 days and maximum of 56 days. These results are different from those that were published in another study where the median hospital length of stay was 9 days, with a minimum of 1 day and maximum of 20 days [12]. In addition, more patients on admission had elevated AST (58.4%) than ALT (39%), in comparison to our results where we did not observe a significant difference between AST and ALT on admission (21.93% patients had elevated AST and 22.81% patients had elevated ALT).

Furthermore, in both studies, transaminases were statistically significantly higher in men than in women [11]; more precisely in our study 41.1% of men had elevated liver enzymes, and 28.3% of women (*p* = 0.017). According to this fact, we could assume that men with elevated transaminases would have a higher CT scan severity score than women, but no correlation between gender and CT severity score in patients with liver damage was observed (*p* = 0.435).

On the other hand, we need to emphasize that the CT scan severity score in our study was statistically significantly higher in patients with AST and/or ALT > 40 U/L than in those with normal levels of transaminases (*p* < 0.05). Patients with elevated transaminases also had a moderate or severe bilateral pneumonia on CT scan of thorax more often (83.7%) in comparison to those without elevated transaminases (64.63%).

In our study, aspartate aminotransferase was the best individual predictor of CT scan severity score (Adjusted R² = 0.299) among others, including the values of neutrophils and leukocytes on admission (Adjusted R² = 0.252). It has already been mentioned that we could predict CT scan severity score according to the values of AST and neutrophils, platelets, and monocytes count on admission. This is in concordance with previously published studies. Xie et al. found that COVID-19 patients with liver damage had higher CT scan severity score, indicating that CT scan severity score was also a predicting factor of liver injury in COVID-19 patients [6].

We had no patients addmited to ICU in our study. However, it was shown that up to 62% of patients admitted to ICU had elevated AST compared to 25% of non-ICU patients [13]. This can also imply that elevated AST is more frequently seen in the severe forms of COVID-19.

As previously noted, liver damage in COVID-19 infection has a multifactorial mechanism. First of all, it is unproven if liver impairment can be provoked directly by the virus itself [14]. What we know until now is that SARS-CoV-2 uses angiotensin-converting enzyme 2 (ACE2) as a receptor to enter the cell [15]. These receptors can be found in type 2 alveolar epithelial cells, in most of the cholangiocytes and in less than 3% of hepatocytes [16], but, despite this, more patients had elevated transaminases [17]. The presence of ACE2 receptors in cholangiocytes also indicates that liver injury can be caused by infection of cholangiocytes [16], as these cells contribute to liver regeneration [18]. One more mechanism of liver damage may be by hepatotoxic drugs, especially antivirals and biological therapy targeting interleukin-6 (IL-6), but also antipyretics and antimicrobials [19,20]. AST is present in both liver and muscle tissue, so myolysis can lead to an increased level of AST [20]. Furthermore, as we know, ACE2 can also be found in epithelial cells of enterocytes in ileum and colon [21,22,23], so due to viral replication in the intestine SARS-CoV-2 RNA is present in patients’ stool [24] and the fecal-oral route could be a possible mode of COVID-19 transmission [25]. Therefore, it is hypothesized that the virus or inflammatory mediators from the intestine can use portal circulation to invade the liver [26]. In addition, Kupffer cells combat the virus by activating an inflammatory response [26]. It can be concluded that a possible mechanism is systemic inflammation made by cytokine storm [19,27]. It is known that COVID-19 leads to massive endothelial dysfunction and widespread coagulopathy. Moreover, in a study observing results after liver biopsies, dilation of portal vein branches, luminal thrombosis, portal tract fibrosis and microthrombi in the sinusoids were reported [28,29]. This implies that coagulopathy can impair liver perfusion and lead to ischemia, and eventually cell death [26]. In another study, post-mortem autopsy liver biopsy revealed moderate microvascular steatosis and mild lobular and portal activity [30]. An important cause of the increased transaminases could also be the ischemia of the liver caused by hypoxia due to respiratory failure in COVID-19 patients [31].

Although we excluded patients with a health record diagnosis of NALFD and patients who had elevated liver enzymes before admission, we must mention that this disease is a more significant risk factor for hospital admission in COVID-19 patients, compared to age, gender, obesity, or other comorbidities [8]. NAFLD implies a pro-inflammatory state which can favor the cytokine storm leading to the multiorgan failure frequently seen in severe COVID-19 infection [8]. As these patients are more prone to have a severe form of this infection, they should be cautiously treated, firstly for their underlying metabolic disease.

The present study has several limitations. The sample size is relatively small having in mind the number of patients that were positive for SARS-CoV-2 and treated in hospital conditions. Further evaluation of the patients treated in ICU is needed as these patients usually have severe forms of the disease leading to larger end-organ damage. The evaluation of liver enzyme values through hospital course regarding the given therapy can also provide important information on the cause of the liver damage. The correlation between these variables and important clinical outcomes is yet to be established.

## 5. Conclusions

SARS-CoV-2 is a systemic disease that mainly affects the respiratory tract, but also leads to liver damage. Liver injury correlates with the severity of COVID-19 infection, which is extremely important in everyday clinical practice. The most significant predictors in this study were the levels of AST, neutrophils, platelets, and monocytes count on admission. These variables could account for almost half (49.5%) of the variability in CT scan severity score at the peak of the disease among cases, predicting the extensiveness of interstitial pneumonia related to COVID-19 infection. Knowing this fact, we can stratify patients with higher risk for the severe forms of COVID-19 in order to provide proper care and start the beforehand therapy in a timely manner. In conclusion, liver enzymes, especially AST as the best single predictor, should be closely monitored in COVID-19 patients, to plan the step-up treatment. Further studies, preferably multi-centric, must be conducted to confirm the results, especially in critically ill patients admitted to the intensive care unit. Additionally, since liver injury in this infection has a multifactorial mechanism, more complex research in future is needed.

## Figures and Tables

**Table 1:** Regression model for CT scan severity score.

Variable on Admission	Beta	Lower	Upper	Sig
Aspartate aminotransferase	0.078	0.047	0.109	0
Neutrophils	2.436	1.126	3.747	0
Leukocytes	−1.911	−3.184	−0.637	0.004

**Table 2:** Regression model for CT scan severity score at peak of infection.

Variable on Admission	Beta	Lower	Upper	Sig
Aspartate aminotransferase	0.111	0.072	0.15	0
Neutrophils	0.847	0.291	1.404	0.004
Platelets	0.025	0.006	0.044	0.012
Monocytes	−10.174	−18.855	−1.493	0.023

## Data Availability

The data that support the findings of this study are available on request from the corresponding author. The data are not publicly available due to privacy and ethical restrictions.
